# Alcohol Misuse and Associations with Childhood Maltreatment and Out-of-Home Placement among Urban Two-Spirit American Indian and Alaska Native People

**DOI:** 10.3390/ijerph111010461

**Published:** 2014-10-14

**Authors:** Nicole P. Yuan, Bonnie M. Duran, Karina L. Walters, Cynthia R. Pearson, Tessa A. Evans-Campbell

**Affiliations:** 1 Mel and Enid Zuckerman College of Public Health, University of Arizona, 1295 North Martin Avenue, P.O. Box 245209, Tucson, AZ 85724, USA; 2 School of Public Health, University of Washington, 1959 NE Pacific Street, Box 357660, Seattle, WA 98195, USA; E-Mail: bonduran@u.washington.edu; 3 Indigenous Wellness Research Institute, School of Social Work, University of Washington, 4101 15th Avenue NE, Box 354900, Seattle, WA 98105, USA; E-Mails: kw5@u.washington.edu (K.L.W.); pearsonc@u.washington.edu (C.R.P.); tecamp@u.washington.edu (T.A.E.-C.)

**Keywords:** alcohol misuse, child maltreatment, out-of-home placement, American Indian, sexual minority

## Abstract

This study examined associations between alcohol misuse and childhood maltreatment and out-of-home placement among urban lesbian, gay, and bisexual (referred to as two-spirit) American Indian and Alaska Native adults. In a multi-site study, data were obtained from 294 individuals who consumed alcohol during the past year. The results indicated that 72.3% of men and 62.4% of women engaged in hazardous and harmful alcohol use and 50.8% of men and 48.7% of women met criteria for past-year alcohol dependence. The most common types of childhood maltreatment were physical abuse among male drinkers (62.7%) and emotional abuse (71.8%) among female drinkers. Men and women reported high percentages of out-of-home placement (39% and 47%, respectively). Logistic multiple regressions found that for male drinkers boarding school attendance and foster care placement were significant predictors of past-year alcohol dependence. For female drinkers, being adopted was significantly associated with a decreased risk of past-year drinking binge or spree. Dose-response relationships, using number of childhood exposures as a predictor, were not significant. The results highlight the need for alcohol and violence prevention and intervention strategies among urban two-spirit individuals.

## 1. Introduction

From 1999–2009, American Indian and Alaska Native (AI/AN) people had higher rates of alcohol-attributable death compared to Whites [1]. Leading chronic causes were alcoholic liver disease, alcohol dependence, and unspecified liver cirrhosis. Despite the burden of alcohol for the AI/AN population, there are few studies with specific sub-groups, including lesbian, gay, bisexual, and transgender (LGBT) people, hereafter referred to as two-spirit individuals. Although not universally accepted among all LGBT AI/AN communities, the term “two-spirit” is generally used across several urban LGBT AI/AN communities [2]. The lack of alcohol research is surprising given documented risks of alcohol dependence among sexual minorities in the general population. For instance, a meta-analysis with the general population found that the risks for alcohol misuse were 1.5 times higher among LGBT individuals compared to heterosexual people [3]. Among the limited research, there is some evidence that alcohol misuse among two-spirit individuals is a public health concern [4,5,6]. For instance, one study conducted in the greater New York metropolitan area found that two-spirit participants, on average, reported that they had their first alcohol drink at a younger age than heterosexual individuals [4]. Two-spirit individuals also had higher scores on two subscales that measured drinking to improve social skills and drinking to manage mood and tension compared to their heterosexual counterparts. However, separate analyses with only the male sample showed no differences in the frequency of 12-month alcohol use between two-spirit and heterosexual men [7]. Given that both analyses were limited to sample sizes of 25 or fewer two-spirit people, additional research is needed with larger community samples.

Little is known about factors associated with alcohol misuse among two-spirit people. Research with general AI/AN communities and general population of sexual minorities have examined the impact of childhood exposures, including different forms of maltreatment. Studies with reservation-based [8] and urban AI/AN samples [9] have documented significant associations between childhood abuse and substance use. National studies with sexual minorities in the general population have also provided evidence of positive associations [10,11]. For instance, the National Epidemiologic Survey on Alcohol and Related Conditions showed that lesbians who experienced childhood neglect had more than 30 times the odds of past-year alcohol dependence compared to heterosexual women with the same childhood experience [10].

Other childhood exposures of interest to AI/AN communities are out-of-home placement, including boarding school attendance, foster care placement, and adoption. All three were used historically to remove AI/AN children from their biological families, extended families, and reservation settings [12]. The Boarding School Era of the 1880’s–1950’s was referred to as the first generation of AI/AN child removal and assimilation policy [13]. The Indian Adoption Era of the 1950’s–1970’s was the second generation of child welfare policy [13]. Between the mid-1800s and the 1930s, thousands of AI/AN children were placed in Indian boarding schools and they were punished if they engaged in cultural and spiritual practices [14]. Most of the schools closed during the 1970s, but a few remained open to educate at-risk AI/AN youth and those who lived in rural areas [14]. Between 1958 and 1967, the Child Welfare League of America and the Bureau of Indian Affairs created the Indian Adoption Project, reducing barriers to interstate adoption and promoting adoption by non-Indian families as the preferred solution for AI/AN children living in poverty [15]. Despite the passage of the Indian Child and Welfare Act (ICWA) of 1978, AI/AN children continued to be disproportionally represented in the U.S. public child welfare systems [16]. An analysis of 2009 national data found that AI/AN children had an out-of-home placement disparity ratio of 3.17 compared to White children [17].

Research on the health effects of out-of-home placement among the indigenous population is limited to a small group of studies that have produced mixed findings. For example, one study found that AI/AN men who attended boarding school were more likely to have a diagnosis of a drug use disorder compared to men who had not attended boarding school [12]. In contrast, a separate study found that Navajo Indians with a history of alcohol dependency were not more likely to have attended boarding school compared to those without alcohol dependency [18]. Similar inconsistent findings have been documented for adoption. Adoption was associated with anxiety disorders among women from a Southwestern tribe [12]. A more recent study conducted with the Inuit people indicated that adoption did not increase the risk of psychiatric admission [19]. Further research is warranted, including studies with two-spirit people who might experience heightened vulnerabilities due to exposure to racism, sexism, and homophobia/heterosexism in out-of-home placement settings.

Because multiple childhood exposures have been documented among the general AI/AN population [8], investigations on the cumulative effects of adversities among two-spirit people are needed. The landmark Adverse Childhood Experiences (ACE) Study measured ten categories of childhood abuse and household dysfunction. The findings pointed to a strong dose-response relationship between number of childhood exposures and risk factors that contributed to morbidity and mortality, including alcoholism, among U.S. adults [20]. Similar analyses were conducted by two studies with the general AI/AN population, both of which reported significant dose-response effects [8,21]. To date, dose-response effects of childhood exposures have yet to be tested among the two-spirit population. The purpose of the current study was to examine patterns of alcohol misuse among two-spirit adults who reported consuming alcohol during the past year and to estimate the impact of specific types and number of different childhood exposures on alcohol misuse. Exposures under investigation were childhood abuse and neglect and out-of-home placement. All models were tested separately by gender because of documented gender differences in alcohol misuse and childhood exposures among the general AI/AN population [8].

## 2. Methods

### 2.1. Recruitment

This study used data obtained from a multi-site, cross-sectional investigation of the health of two-spirit AI/AN people, referred to as the HONOR Project. Participants were recruited from seven U.S. metropolitan sites including Seattle-Tacoma, San Francisco-Oakland, Los Angeles, Denver, Tulsa-Oklahoma City, Minneapolis-St. Paul, and New York [22]. Individuals who were eligible to participate in the main study were: (1) self-identified as American Indian, Alaska Native, or First Nation and enrolled in a tribal nation or reported having at least 25% American Indian blood; (2) self-identified as gay, lesbian, bisexual, transgender, or two-spirit (GLBTT-S) or engaged in same-sex sexual behaviors in the past 12 months; (3) 18 years or older; (4) English speaking; and (5) residing, working, or socializing in one of the urban study sites.

Three recruitment methods were used to maximize coverage of the heterogeneity of the population and to minimize selection bias. They included targeted, partial-network, and respondent-driven sampling techniques (for further details see Chae and Walters [22]). Targeted sampling consisted of having study coordinators identify 6 to 8 initial recruits, referred to as “seeds,” based on their relationships to GLBTT-S people in each participating metropolitan area. A total of 36 first wave seeds were recruited, out of which 33 participated in the study. Then a partial network technique was implemented, requesting all participants to provide information about individuals in their social networks, referred to as “nominees,” who might be eligible to participate in the study. For each nominee who contacted the project team, the participant received $10 (identity of the “nominee” was protected). Out of the 58 nominees, 50 participated in the study. At a census site, an additional 469 individuals, referred to as “volunteers,” were recruited through newsletters, brochures, posters, and word of mouth, out of whom 368 participated [23]. There were no significant differences between participants who were identified by respondent-driven approaches (seeds and nominees) and volunteers on major sociodemographic variables that might indicate regional or sampling differences [22].

### 2.2. Sample

A total of 451 participants completed interviews during the study period between July 2005 and March 2007. Four participants were later excluded from the analytic dataset because they did not meet eligibility criteria, resulting in a sample size of 447 participants. For the current study, analyses were conducted with 294 participants who self-reported consumption of some type of drink containing alcohol during the past 12 months. Individuals who were lifetime abstainers (n = 7) and those who did not consume alcohol in the past year (n = 111) were excluded. Individuals who self-identified as transgender (n = 35) were also excluded from the analytical dataset due to the small sample size and focus on gender comparisons.

### 2.3. Procedure

Participants completed a 3–4 hour self-administered computer-assisted interview in a private location of their choice. Trained interviewers were available to answer questions and provide assistance while respecting participants’ privacy [24]. Almost all of the interviewers were AI/AN or two-spirit and were from diverse socioeconomic backgrounds. The interviewers received extensive training before beginning data collection. All participants provided written informed consent prior to the start of the study and received compensation of $65 at the completion of the interview. Data collection began after receiving approval from the Institutional Review Board at the University of Washington.

### 2.4. Measures

The structured interview consisted of standardized instruments, measures adapted for the AI/AN population, and instruments created for the main study. The present study analyzed sections of the interview that assessed sociodemographic characteristics, alcohol misuse, and childhood exposures.

*Sociodemographic characteristics.* Sociodemographic variables used in the analyses included gender, age, education level, and household monthly income.

*Alcohol misuse.* Alcohol misuse during the past year was investigated using three separate measures. Diagnoses of alcohol disorders were determined using the Mini-International Neuropsychiatric Interview (M.I.N.I. version 5.0) [25]. Past-year alcohol abuse required one out of four diagnostic criteria during the past 12 months. Past-year alcohol dependence was based on the presence of at least three of seven diagnostic criteria during the past 12 months. Each criterion was assessed with a single item scored as yes/no. The M.I.N.I. has been shown to have high validity and reliability compared to other scheduled interviews [26,27]. Hazardous and harmful alcohol consumption was assessed using the Alcohol Use Disorders Identification Test (AUDIT) [28]. The AUDIT is a 10-item instrument that covers alcohol consumption, drinking behavior, and alcohol-related problems [29]. Responses to each question are scored from 0 to 4, with a total possible score of 40. Total raw scores were coded as dichotomous variables using the established cut-point of 8 as an indicator of hazardous and harmful alcohol use. In a review article, median sensitivity was 0.86 and median specificity was 0.89 across studies [30]. Drinking binge or spree was assessed using a single item. Participants were asked, “In the last 12 months, did you go on a drinking binge or spree in which you stayed drunk for two whole days or more?” Responses were provided as yes/no.

*Childhood exposures*. Eight categories of childhood exposures were assessed. Five categories of childhood maltreatment consisted of physical abuse, physical neglect, sexual abuse, emotional abuse, and emotional neglect. They were measured using the short form of the Childhood Trauma Questionnaire (CTQ-SF) [31]. The CTQ-SF is a 28-item Likert-scaled questionnaire that assesses abuse and neglect before the age 18. For instance, participants rated the statement “When I was growing up, I didn’t have enough to eat.” The current study used a 6-point scale. The responses were converted to a 5-point scale in order to calculate subscale scores consistent with the original CTQ-SF. Each converted subscale score ranged from 5 (no history abuse and/or neglect) to 25 (extreme history of abuse and/or neglect). Empirically-validated cut scores were applied to categorize individuals with histories of abuse and neglect [32]. The cut-off points were: physical abuse (8), physical neglect (8), sexual abuse (8), emotional abuse (10), and emotional neglect (15). Three categories of out-of-home placement included Indian boarding school attendance, foster care placement, and adoption. Respondents were asked, “Did you ever attend an Indian boarding school,” “Were you in foster care?” and “Were you legally adopted?” Yes/no responses were provided for each question.

### 2.5. Statistical Analysis

All analyses were conducted using SPSS 20 (IBM Corporation, Somers, NY, 2011). Percentages of past-year alcohol misuse, childhood maltreatment, and out-of-home placement were estimated and male/female gender comparisons were conducted using Chi-square tests. Separate logistic regression models were tested for each past-year alcohol outcome variable, including alcohol dependence, hazardous and harmful alcohol consumption, and drinking binge or spree for two days or more. Using bivariate analyses, each type of childhood exposure was assessed for associations with each alcohol outcome variable. Subsequent multivariate logistic regressions tested childhood exposures that were significant predictors (*p* < 0.05) in the initial models. Dose-response analyses were conducted using number of types of childhood exposures (range 0–8) as the predictor of past-year alcohol dependence, the most severe drinking problem assessed in the study. All regression models were adjusted for education level and household income because of significant associations with alcohol variables at the bivariate level. All models were tested separately for men and women.

**Table 1:** Demographic characteristics of study participants (N = 294).

Variable	Men (n = 177) N (%)	Women (n = 117) N (%)
Age (M, SD, range)	37.97 (10.54, 20–63)	38.98 (10.42, 18–62)
Education		
Not graduated HS	20 (11.3)	28 (23.9)
Graduated HS/GED	60 (33.9)	34 (29.1)
Post HS but no degree	36 (20.3)	20 (17.1)
Vocational/Associate	28 (15.8)	16 (13.7)
Bachelor degree	18 (10.2)	12 (10.3)
Graduate/profess degree	15 (8.5)	7 (6.0)
Currently employed (part or full-time)	79 (44.6)	41 (35.0)
Monthly income		
$0–1000	86 (50.0)	70 (62.5)
$1001–2000	36 (20.9)	14 (12.5)
$2001–5000	34 (19.8)	20 (17.9)
$5001 or more	16 (9.3)	8 (7.1)
Missing data	5 (2.8)	5 (4.3)
Currently enrolled member of a tribal nation	35 (76.3)	90 (76.9)
Primary setting where raised		
Reservation/tribal lands	50 (28.2)	17 (14.5)
Off-res in urban areas	56 (31.6)	47 (40.2)
Off-res in suburban areas	29 (16.4)	23 (19.7)
Off-res in rural areas	25 (14.1)	17 (14.5)
Other	17 (9.6)	13 (11.1)

## 3. Results

### 3.1. Sample Demographics

As indicated in Table 1, 177 (60.2%) participants were male. Among men, the average age was 38 years (range 20–63), 88.7% were high school graduates, and 28.2% had a household monthly income of $2001 or higher. Among women, the average age was 39 years (range 18–62), 76.1% were high school graduates, and 23.9% had a household monthly income of $2001 or higher.

### 3.2. Alcohol Misuse

Among male current drinkers, 14.1% were diagnosed with past-year alcohol abuse and 50.8% were diagnosed with past-year alcohol dependence (Table 2). A larger proportion (72.3%) met criteria for hazardous and harmful alcohol use in the past year. More than a third (38.4%) reported having a drinking binge or spree for two whole days or more during the past year. Among female current drinkers, 12% were diagnosed with past-year alcohol abuse and 48.7% were diagnosed with past-year alcohol dependence. A larger proportion (62.4%) met criteria for hazardous and harmful alcohol use in the past year. Forty-one percent reported drinking binge or spree for two whole days or more during the past year. A significantly greater number of men met criteria for past-year hazardous and harmful alcohol use compared to women (*p* < 0.05).

**Table 2:** Percentages of alcohol misuse, childhood maltreatment, and out-of-home placement.

Variable	Total Sample (n = 294) N (%)	Men (n = 177) N (%)	Women (n = 117) N (%)	Test Statistic
Past-year alcohol misuse				
Alcohol abuse	39 (13.3)	25 (14.1)	14 (12)	0.59
Alcohol dependence	147 (50)	90 (50.8)	57 (48.7)	1.64
Hazardous and harmful alcohol use ^a^	201 (68.4)	128 (72.3)	73 (62.4)	4.00 *
Drinking binge or spree for two whole days or more	116 (39.5)	68 (38.4)	48 (41)	0.14
Childhood maltreatment ^b^				
Physical abuse	191 (65)	111 (62.7)	80 (68.4)	1.16
Physical neglect	191 (65)	109 (61.6)	82 (70.1)	3.70
Sexual abuse	172 (58.5)	90 (50.8)	82 (70.1)	12.04 **
Emotional abuse	191 (65)	107 (60.5)	84 (71.4)	5.91 *
Emotional neglect	115 (39.1)	61 (34.5)	54 (46.2)	4.67 *
Out-of-home placement				
Indian boarding school	53 (18)	30 (16.9)	23 (19.7)	0.35
Adoption	40 (13.6)	23 (13.0)	17 (14.5)	0.15
Foster care	74 (25.2)	34 (19.2)	40 (34.2)	8.39 **

Notes: ^a^ Individuals who had a total score of 8 or higher on the AUDIT were categorized as engaging in hazardous and harmful alcohol use; ^b^ Individuals who scored at or above threshold scores for each Childhood Trauma Questionnaire subscale were assigned to each maltreatment group. Gender comparisons were conducted using Chi-square tests. Test statistics were derived using Pearson’s Chi-square. * *p* < 0.05; ** *p* < 0.01.

### 3.3. Childhood Maltreatment and Out-of-Home Placement

There was a high percentage of childhood maltreatment among two-spirit AI/AN people (Table 2). Among men who drank during the past year, the most common types of child maltreatment were physical abuse (62.7%), physical neglect (61.6%), and emotional abuse (60.5%). Among women who drank during the past year, the most common types of child maltreatment were emotional abuse (71.8%), physical neglect (70.1%), and sexual abuse (70.1%). Women reported significantly higher rates of childhood sexual abuse, emotional abuse and emotional neglect compared to men (*p* < 0.05). Both men and women reported high percentages of being placed out of the home during childhood (39% and 47%, respectively). Women reported significantly higher percentages of foster care placement compared to men (34.2% *vs.* 19.2%; *p* < 0.01).

### 3.4. Relationships between Single Childhood Exposures and Alcohol Misuse

Bivariate logistic regressions showed significant associations between childhood exposures and alcohol misuse (Table 3). For men, attending an Indian boarding school and being placed in foster care were each significantly associated with a greater than three-fold increase risk of past-year alcohol dependence. Indian boarding school attendance was also associated with a greater than three-fold increase risk of past-year hazardous and harmful alcohol use and a six-fold increase risk of past-year drinking binge or spree for two or more days. For women, associations between single types of childhood exposures and past-year alcohol dependence and hazardous and harmful alcohol use were not significant (Table 3). Experiencing childhood physical neglect and emotional abuse were significantly associated with increased risks of past-year drinking binge or spree for two or more days. Being adopted was significantly associated with *decreased* risk of past-year drinking binge or spree for two or more days.

### 3.5. Relationships between Multiple Childhood Exposures and Alcohol Misuse

For men, a multivariate regression model was only conducted for past-year alcohol dependence. The other two alcohol variables did not have more than one significant predictor (*p* < 0.05) identified in initial models. When both were entered in a multivariate regression model, Indian boarding school attendance (OR = 3.34, 95% CI = 1.28, 8.75, *p* < 0.05) and foster care placement (OR = 2.99, 95% CI = 1.19, 7.53, *p* < 0.05) remained significant predictors of past-year alcohol dependence. For women, a multivariate regression model was conducted for past-year drinking binge and spree due to multiple significant predictors identified in initial models. When three significant predictors were entered in the model, only being adopted was significantly associated with past-year drinking binge and spree (OR = 0.12, 95% CI = 0.02, 0.60, *p* < 0.05).

**Table 3:** Single childhood exposures and adjusted odds ratios (OR) of past-year alcohol misuse.

Childhood Exposures	Men			Women		
	Alcohol dependence	Hazardous and harmful use	Drinking binge/spree	Alcohol dependence	Hazardous and harmful use	Drinking binge/spree
	OR 95% CI	OR 95% CI	OR 95% CI	OR 95% CI	OR 95% CI	OR 95% CI
Childhood maltreatment						
Physical abuse	1.39 0.71, 2.72	1.03 0.47, 2.23	1.10 0.54, 2.23	1.27 0.45, 3.54	3.07 0.98, 9.58	2.54 0.78, 8.25
Physical neglect	1.50 0.75, 3.00	0.95 0.43, 2.07	1.39 0.67, 2.90	2.62 0.92, 7.46	2.31 0.81, 6.55	4.58 1.27, 16.48 *
Sexual abuse	1.05 0.55, 2.00	0.64 0.30, 1.35	0.79 0.39. 1.58	0.90 0.32, 2.55	0.73 0.23, 2.37	2.28 0.70, 7.42
Emotional abuse	1.27 0.65, 2.46	0.86 0.40, 1.86	0.91 0.45, 1.84	1.53 0.57, 4.13	1.55 0.54, 4.45	3.93 1.22, 12.62 *
Emotional neglect	1.49 075, 2.96	0.96 0.44, 2.11	1.29 0.63, 2.66	0.45 0.18, 1.13	0.45 0.16, 1.24	0.58 0.22, 1.53
Out-of-home placement						
Boarding school	3.81 1.48, 9.82 **	3.53 1.05,11.81 *	6.04 2.25, 16.21 **	1.54 0.54, 4.44	2.35 0.65, 8.44	2.01 0.63, 6.46
Adoption	1.22 0.46, 3.22	0.69 0.23, 2.01	1.37 0.51, 3.66	1.25 0.37, 4.15	1.22 0.34, 4.36	0.16 0.03, 0.71 *
Foster care	3.46 1.39, 8.62 **	1.23 0.45, 3.35	2.24 0.94, 5.34	0.91 0.35, 2.38	1.33 0.46, 3.87	1.04 0.38, 2.86

Notes: OR = odds ratio; CI = confidence interval. Odds ratios were adjusted for education level and household income. * *p* < 0.05; **p < 0.01.

**Table 4:** Percentages of number of childhood exposures and adjusted odds ratios (OR) of past-year alcohol dependence.

Number of Exposures	Total N (%)	Men			Women			Test Statistic
		N (%)	OR	95% CI	N (%)	OR	95% CI	
0	25 (8.5)	18 (10.2)		Reference	7 (6)		Reference	1.48
1	36 (12.2)	28 (15.8)	1.81	0.48, 6.85	8 (6.8)	0.79	0.04, 15.87	5.07*
2	31 (10.5)	19 (10.7)	0.59	0.14, 2.59	12 (10.3)	4.75	0.31, 72.87	0.01
3	33 (11.2)	24 (13.6)	1.55	0.39, 6.16	9 (7.7)	1.63	0.09, 30.23	2.29
4 or more	162 (55.1)	85 (48)	2.04	0.68, 6.14	77 (65.8)	1.91	0.17, 21.61	10.37 **

Notes: Odds ratios were adjusted for education level and household income. Test statistics were derived using Pearson’s Chi-square. * *p* < 0.05; ** *p* < 0.01.

### 3.6. Dose-Response Effects of Childhood Exposures

The majority of men and women experienced one or more types of childhood exposures (88.1% and 90.6%, respectively; Table 4). Most participants reported four or more childhood exposures (men: 48%; women: 65.8%). Gender differences were significant among participants who reported experiencing one type of childhood exposure and those who reported experiencing four or more types. For both men and women, dose-response relationships between the number of childhood exposures and past-year alcohol dependence were not significant.

## 4. Discussion

### 4.1. Findings

The current findings identified alcohol misuse as a public health concern for some urban two-spirit American Indian and Alaska Native people. Among two-spirit AI/AN men who consumed alcohol during the past year, 50.8% were diagnosed with past-year alcohol dependence. The past-year percentage was higher than those documented among AI/AN men from two reservation communities (8.9% and 12.7%) [33]. Two-spirit female drinkers also had substantially higher percentage of past-year alcohol dependence (48.7%) compared to AI/AN women from the same two-reservation study (1.1% and 7.0%). Greater percentages of two-spirit men (72.3%) and women (62.4%) met criteria for past-year hazardous and harmful alcohol use than those diagnosed with alcohol dependence in the current study. Past-year drinking binge or spree was also common among the sample.

High percentages of childhood exposures were also documented among urban two-spirit AI/AN people. Among two-spirit male current drinkers, percentages of childhood physical abuse, physical neglect, and emotional abuse were each greater than 60%. Percentages were substantially higher than those documented for men in a multi-tribal, reservation-based study [8]. Among two-spirit female current drinkers, percentages of physical neglect, sexual abuse, and emotional abuse were each about 70% and also much higher than those among AI/AN women from the same multi-tribal study. Reasons for the heightened risks of childhood maltreatment among two-spirit AI/AN people are unknown. One explanation is that two-spirit children and adolescents may be more vulnerable to violence victimization because they have dual or triple oppressed statuses. They might experience racism, sexism, and homophobia/heterosexism from different groups, including AI/AN people, non-AI/AN people, and sexual minorities [34]. Future research should examine contextual factors of childhood maltreatment among AI/AN sexual minorities, including co-occurring exposures to prejudice, discrimination, and harassment, and the impact on health.

Two-spirit men and women reported high percentages of out-of-home placement (39% and 47%, respectively), with foster care being the most common type of experience. Out-of-home placement may be associated with childhood maltreatment. Many AI/AN children who were in the child welfare system were placed because of evidence of child abuse and neglect. Previous analyses with the HONOR Project dataset showed that several participants experienced physical or sexual abuse while attending boarding schools [5]. Future studies on the temporal relationship between childhood maltreatment and out-of-home placement may identify important targets for prevention and child welfare policies benefiting AI/AN communities.

The lack of significant associations between childhood maltreatment and alcohol misuse among two-spirit AI/AN people was surprising. The findings may be due to temporal proximity to current drinking behaviors. A reservation-based study found that the effect of distal adversity, including childhood trauma, on substance dependence onset was less evident when proximal adversities were present [21]. Proximal adversities were not investigated in the current study, but might have influenced alcohol misuse among the two-spirit AI/AN sample. Future research should examine proximal factors, including discrimination based on race, gender, and sexuality [22]. Past analyses with the HONOR Project dataset showed that discrimination was associated with physical pain and impairment and fair or poor health [22]. Research with the general U.S. population found that lesbian, gay, and bisexual adults who reported three types of discrimination based on race, gender, and sexual orientation were 4 times more likely to have any past-year substance use disorder compared to those who did not report discrimination [35].

A noteworthy finding was that Indian boarding school attendance and foster care placement were associated with alcohol misuse among two-spirit men, but not women. Past findings with reservation-based communities have been mixed [8,12,18] and underlying mechanisms have not yet been identified. In the current study, it was not possible to determine if the gender differences were attributed to differences in actual experiences or perceptions of and meanings associated with Indian boarding schools. Some AI/AN people have reported positive boarding school experiences, including opportunities to build skills [36] and removal from dysfunctional home environments [37]. The two-spirit men in the present study might have assigned different meanings to their experiences of boarding school and foster care compared to the two-spirit women. They might have viewed boarding school experiences within historical contexts that contributed to increased levels of distress and risk of alcohol misuse. Boarding schools were often a metaphor for colonialism because of the removal of children from their parents and suppression of AI/AN culture [38]. The boarding school movement was related to historical trauma, defined as massive group trauma that contributes to cumulative emotional and psychological distress across the lifespan and multiple generations [39]. Research has shown that having parents, grandparents, or caregivers who attended boarding schools increased the risks of mental health concerns among younger generations of indigenous people [5,40]. The lasting negative impact of out-of-home placement experiences may also be linked to heightened vulnerability to stressors later in life [41]. Similar to findings with other racial minority groups [42], two-spirit adults may be exposed to dual levels of maltreatment and stigma due to being both a racial and sexual minority. It was unknown if there were gender differences in recent stressors among the current two-spirit sample and if the stressors reinforced the impacts of childhood exposures among two-spirit men.

Another explanation focuses on the strengths and resiliency of the two-spirit female participants who experienced out-of-home placement, but were not at increased risk of alcohol misuse. It is known that some individuals returned to reservations afterward and embraced tribal culture, language, and community, resulting in family narratives of persistence [38]. Research has documented that AI/AN women who survived trauma might have benefited from spirituality and traditional health practices, enculturation [43] and commitment to tribal community [44]. Two-spirit women obtained strength from family and community, enabling them to embrace their identity more fully [45]. In the current study, sources of strength and support might have reduced the negative effects of out-of-home placement among the sample of two-spirit women, but investigation of this explanation was not possible.

The finding that being adopted was significantly associated with *decreased* risk of past-year drinking binge and spree among two-spirit women was based on a sample of 17 people, and thus, should be interpreted with caution. However, the result provided further evidence of the complexity of out-of-home placement experiences, and suggested that adoption might not be an adverse experience for all AI/AN people. This was consistent with findings from a study conducted in Greenland, which is largely made up of Inuit people [19]. The researchers found that being an adoptee did not increase the risk of psychiatric admission. They proposed that the adoptee’s health was not influenced by the birth origin or adoption, but instead by how the adoptive family, educators, public health, and the society cared for the child and managed adoption-related concerns. The explanation may also be applied to the two-spirit population, but empirical testing is needed.

### 4.2. Implications

The findings have several implications for public health practice. The high rates of alcohol misuse among two-spirit people highlight the need for alcohol screening and brief intervention in settings that are frequently utilized by this population. Single-question screenings are recommended as time-efficient tools that reduce barriers to utilization attributed to multiple-question screens that require scoring [46]. A single screening question for past year heavy alcohol use has been validated among the general population in clinical settings [47]. However, unlike the CAGE questionnaire [48] and CRAFFT [49], single-item alcohol screening tools have not yet been validated with the AI/AN population. In addition, more research is needed on the effectiveness of brief alcohol intervention strategies among AI/AN people. The Screening, Brief Intervention, and Referral to Treatment (SBIRT) protocol may be a useful approach to increase the number of AI/AN people receiving substance abuse treatment [50]. A study using SBIRT at multiple healthcare sites found that it effectively reduced heavy alcohol use at a 6-month follow-up among a subsample of AI/AN people [51].

The high rates of child abuse and neglect call attention to the need for violence prevention among AI/AN communities, including those located in urban areas. Urban AI/AN communities may benefit from public health strategies that have been adapted for the general AI/AN population and emphasize cultural and historical contexts at all levels of influence [52]. Intervention approaches used in Indian country include education on traditional parenting, community education on violence prevention, support programs that foster interactions across multiple generations, culturally-appropriate shelters, job skills training, and gathering places for modern and traditional cultural activities and practices [52]. The approaches may need to be tailored for multi-tribal communities and other unique characteristics of the urban AI/AN population. Unique characteristics include the tendency to rarely live in localized urban neighborhoods, often residing beyond the city limits and into broader urban areas [53]. The urban AI/AN population is very mobile, frequently moving within the same county and between different counties [54]. Urban AI/AN people also often travel between reservations and urban areas [55]. Such characteristics suggest that special consideration may be needed for the dissemination of violence prevention education and intervention programs for AI/AN people living in urban areas.

The long-lasting effects of out-of-home placement among two-spirit men point to the advantages of assessment of a wider range of exposures, including out-of-home placement, other forms of historical or cultural trauma, and discrimination, to identify meaningful areas for healing and recovery [4]. The findings also support the need for interventions. Developing culturally-appropriate interventions is particularly important because there is evidence that AI/AN people perceive mental health services to be ineffective [56,57]. Some attribute the ineffectiveness to the lack of consistency with AI/AN cultures and perceptions of interventions as contemporary forms of Western colonization [58]. Practitioners who work with two-spirit people may utilize alternative approaches. For example, they may address an individual’s existence within different contexts [58], including exploring the significance of out-of-home placement experiences within the historical context of policies and programs that promoted the genocide and assimilation of AI/AN people. Another approach consists of a relationally-based AI/AN perspective of well-being and emphasizes the interconnections between spirituality, physical status, cognitive and emotional processes, and the environment [58]. Areas of the mind may be strengthened by storytelling and memories of past events in which problems were resolved successfully to instill hope about overcoming current challenges [58]. Regardless of the specific approach, all prevention and intervention efforts should incorporate two-spirit issues in order to create safe places for healing and prevent exposure to additional trauma and discrimination [6]. Practitioners may identify opportunities for cultural education and skills-building to enhance “bicultural” competence among two-spirit people and enable them to manage multiple identities and roles while maintaining good physical and mental health [59]. Future research is needed to determine the effectiveness of culturally-relevant approaches for two-spirit communities and the AI/AN population in general.

### 4.3. Limitations

The current study had some limitations. Causal relationships between childhood exposures and adult alcohol use could not be inferred due to the cross-sectional design. However, temporal relationships were examined using childhood maltreatment that occurred before age 18 and alcohol behavior during the past 12 months. The types of out-of-home placement were also specific to the experiences of children. The findings also do not generalize to two-spirit AI/AN individuals living in rural areas or other racial and sexual minority groups [22]. Two-spirit people living in urban settings may have unique sociodemographic characteristics, life stressors, family and community practices, health behaviors, and AI/AN identity development [60]. Although this study examined multiple measures of alcohol misuse, a more comprehensive assessment of alcohol use behaviors and consequences may have provided greater insights into the long-term impact of childhood exposures. A study on victimization among sexual minority women from the general population used multiple measures of hazardous drinking, including heavy drinking, heavy episodic drinking, intoxication, negative drinking consequences, and symptoms of potential dependence [10]. In addition, the current study did not measure some childhood exposures used in past research, including parental substance use [8,20] and domestic violence, mental illness, and criminal behavior in the household [20]. Those types of exposures might have lasting negative consequences among two-spirit people. The study was also unable to explore the diverse contexts of out-of-home placement, and determine if adoption was a qualitatively different experience from boarding school attendance and foster care among AI/AN children. Also, stratified analyses by sexual orientation were not conducted due to small sample sizes. The usefulness of such analyses have been documented by research that found that bisexual women, but not lesbian women, were more likely to report binge drinking compared to heterosexuals [61].

## 5. Conclusions

The current findings contributed to the growing body of knowledge on alcohol misuse and childhood exposures among urban two-spirit AI/AN adults. Research is needed to examine mediators and moderators of the relationships between childhood exposures and alcohol misuse, with an emphasis on historical and contemporary cultural contexts affecting AI/AN communities. Greater attention also needs to be directed to protective factors and resiliency that may inform culturally-relevant interventions. Shifting away from deficit-focused models and embracing indigenous knowledge and decolonizing frameworks may help end the cycle of discrimination and oppression experienced by two-spirit AI/AN people.
